# Transient domoic acid excitotoxicity increases BDNF expression and activates both MEK- and PKA-dependent neurogenesis in organotypic hippocampal slices

**DOI:** 10.1186/1471-2202-14-72

**Published:** 2013-07-17

**Authors:** Anabel Pérez-Gómez, R Andrew Tasker

**Affiliations:** 1 Department of Biomedical Sciences, University of Prince Edward Island, Charlottetown, PEI, Canada

**Keywords:** Neurotoxicity, Neurogenesis, Kainate receptor agonist, Hippocampus, Organotypic cultures, Brain derived neurotrophic factor, Neurogenesis, PD98059, KN93, H89

## Abstract

**Background:**

We have previously reported evidence of cell proliferation and increased neurogenesis in rat organotypic hippocampal slice cultures (OHSC) after a transient excitotoxic injury to the hippocampal CA1 area induced by low concentrations of the AMPA/kainate agonist domoic acid (DOM). An increased baseline rate of neurogenesis may contribute to recovery from DOM-induced mild injury but the intracellular mechanism(s) responsible for neuronal proliferation remain unclear. The current study investigated the key intracellular pathways responsible for DOM-induced neurogenesis in OHSC including the effects of transient excitotoxicity on the expression of brain-derived neurotrophic factor (BDNF), a well-known regulator of progenitor cell mitosis.

**Results:**

Application of a low concentration of DOM (2 μM) for 24 h followed by recovery induced a significant and long lasting increase in BDNF protein levels expressed by both neurons and microglial cells. Furthermore, the mild DOM toxicity stimulated both PKA and MEK-dependent intracellular signaling cascades and induced a significant increase in BDNF- transcription factor CREB activation and BDNF-receptor TrkB expression. Coexposure to specific inhibitors of PKA and MEK phosphorylation resulted in a significant decrease in the neurogenic marker doublecortin.

**Conclusions:**

Our results suggest that transient excitotoxic insult induced by DOM produces BDNF and CREB overexpression via MEK and PKA pathways and that both pathways mediate, at least in part, the increased neural proliferation resulting from mild excitotoxicity.

## Background

Domoic acid (DOM) is an AMPA/kainate receptor ligand that elicits a very rapid and potent neurotoxic response, and as such, has been used as a reliable research tool to investigate excitotoxic damage *in vivo *[1-4] and *in vitro *[5-7]. The hippocampus, among other brain regions, has been identified as a specific target site having high sensitivity to DOM-induced toxicity [8,9] and, at lower doses, to DOM-induced structural plasticity relevant to temporal lobe epilepsy [10,11]. We have previously reported that mild excitotoxicity produced by low concentrations of DOM was reversible and accompanied by a corresponding increase in the baseline rate of neurogenesis in organotypic hippocampal slice cultures (OHSC) [7]. However, the intracellular mechanisms responsible for cell proliferation and neurogenesis after transient excitotoxic insult remain unclear.

BDNF is a member of the neurotrophin family that plays important roles in many developmentally regulated processes, such as cell survival, differentiation and synaptic plasticity of neurons as well as neurogenesis. Some studies reveal that different forms of excitatory cellular stimulation can enhance BDNF synthesis and secretion [12-14] and, accordingly, low doses of DOM during postnatal development have been proven to induce significant increases in hippocampal BDNF expression as well as in its high-affinity receptor, the tropomyosin-related kinase B (TrkB) in the resulting adult animals [10,11]. One of the most well know transcriptional regulators of BDNF gene expression is the cyclic AMP responsive element binding protein (CREB); activation of which can be mediated by the cAMP-dependent protein kinase (PKA), the mitogen-activated protein kinase (MAPK) pathway or the Ca^2+^/calmodulin-dependent protein kinases (CaMKs), among others, depending on the activating signal and cell type [15-17]. These kinases have been reported to mediate cell proliferation and neurogenesis as well as neurite outgrowth, synaptic transmission and neuronal survival in a number of model systems [18-25] and specifically to promote hippocampal neurogenesis both *in vivo *[26,27] and *in vitro *[28,29].

OHSC preserve normal hippocampal anatomical structure and functional properties *in vitro* for several weeks [30] and provide an alternative model to the hippocampus *in vivo* that is accessible to extensive manipulation [31]. As all types of neurons and glia are preserved with their specific morphologies and localizations, the hippocampal neuronal network organization is very similar to that of the living animal [32-34]. Accordingly, in the current experiments we tested the hypothesis that transient exposure to a low concentration of DOM would enhance BDNF expression in cultured hippocampal slices. Further, we aimed to utilize this *in vitro* system to investigate the activation of key intracellular pathways mediating neuronal proliferation after a mild excitotoxic insult.

## Results

### DOM induced overexpression of BDNF and TrkB

**Figure 1:**
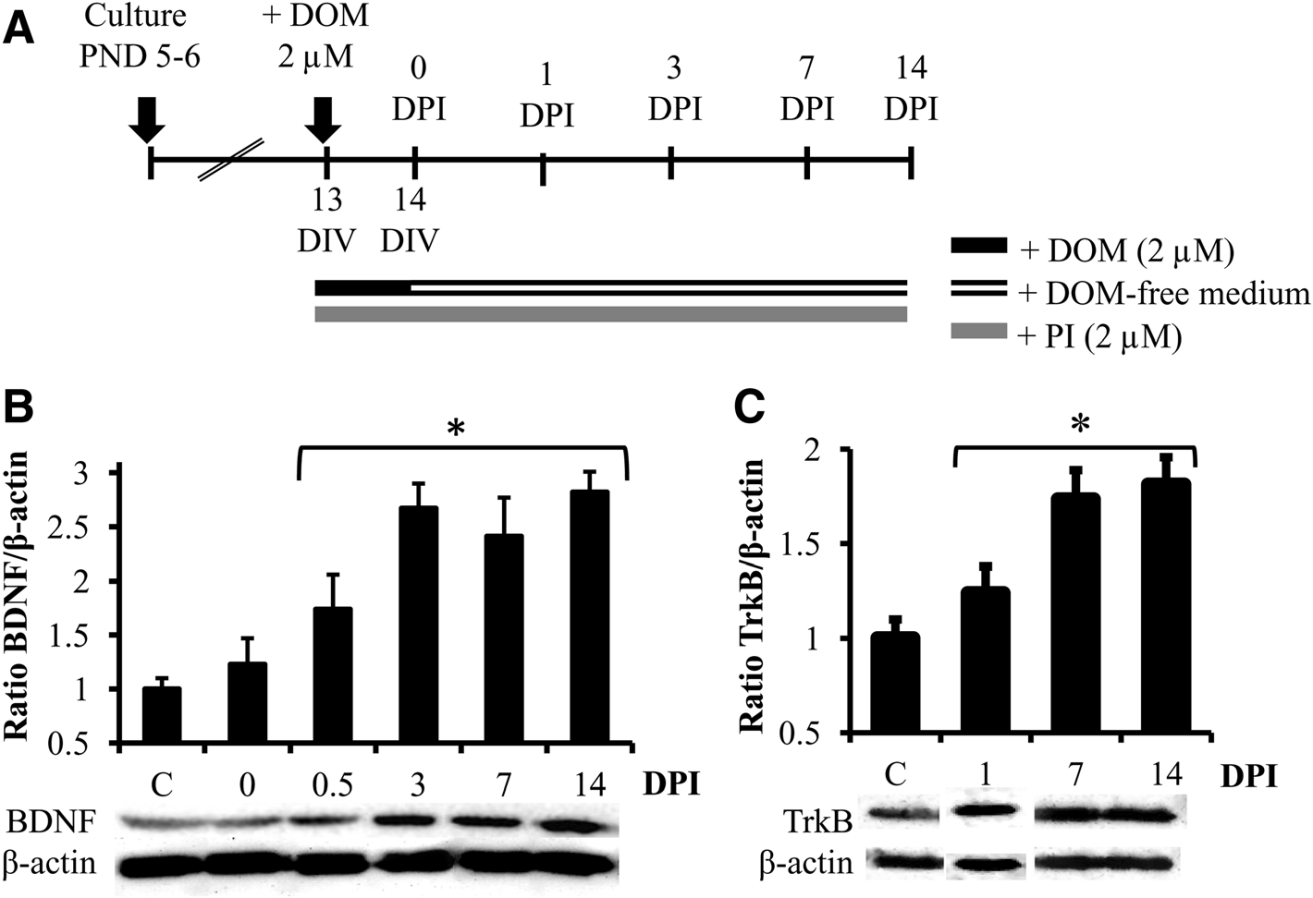
**Transient DOM exposure induces both BDNF and TrkB over-expression in OHSC. (A)** Experimental design and timeline: 13 days-in-vitro (DIV) slices were treated with 2 μM DOM for 24 h (insult) and then changed to a DOM-free medium. Protein samples were collected at the indicated days post insult (DPI). The fluorescent marker of cellular damage propidium iodide (PI, 2 μM) was present in the medium during and after DOM treatment. **(B**,** C)** The effects of transient DOM exposure on BDNF and TrkB expression at different time-points post-insult were analyzed by immunoblot as described in Materials and Methods. The blots correspond to representative experiments and values are the means ± SEM of at least three experiments performed from different cultures (*P < 0.05 vs. C).

To examine whether DOM treatment increases BDNF expression in OHSC, preparations were treated with 2 μM DOM for 24 h (insult), changed to a DOM-free medium and subjected to immunoblot analysis at different times after exposure as summarized in Figure 1A. No significant changes in BDNF levels were found immediately after DOM insult (Figure 1B); however, 12 h post-insult, BDNF levels were significantly increased (~1.5 fold) as compared with non-treated slices. DOM treatment induced a maximum increase in BDNF expression 3 days post-insult (~2.7 fold) compared to age-matched control slices and this increase was maintained up to 14 days post-insult (Figure 1B).

Because BDNF signals primarily through its high-affinity receptor TrkB, expression levels of the TrkB protein were measured in both control and DOM-treated OHSC (Figure 1C). DOM insult led to a sustained increase in the expression of TrkB that was first detected at 24 hours post insult and was sustained throughout the 14 day period (~1.8 fold).

**Figure 2:**
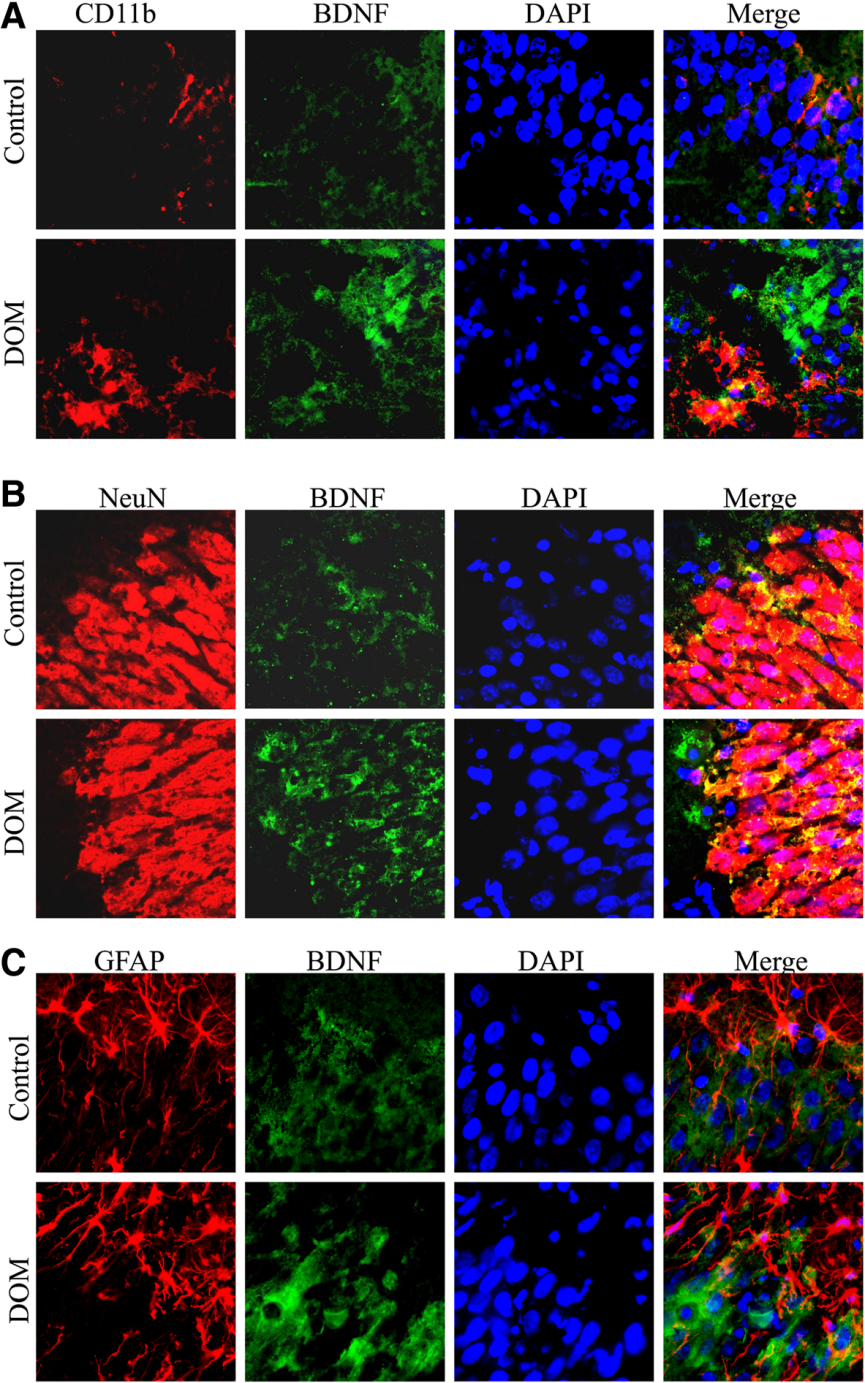
**Immunohistochemical visualization of BDNF in microglia, neurons and astroglial cells in CA1 area, following excitotoxic insult. (A)** Representative fluorescence photomicrographs of CD11b-positive microglial cells (red), BDNF (green), DAPI and merge images. Upper row: control culture, in which BDNF-positive microglial cells have resting-like morphology. Lower row: culture exposed to DOM for 24 h and then transferred to a DOM-free medium for 7 days showed highly activated and BDNF-expressing microglia (lower left quadrant). **(B)** Representative fluorescence photomicrographs of NeuN-positive neurons (red), BDNF (green), DAPI and merge images. Most BDNF immunoreactivity in both control and DOM-treated group was observed in NeuN-positive cells. **(C)** Representative photomicrographs of GFAP-positive astroglial cells (red), BDNF (green), DAPI and merge images. No significant changes were observed in the number of astrocytes expressing BDNF in presence or absence of the DOM insult.

To determine which cell types overexpressed BDNF following transient DOM treatment, we performed double immunostaining for BDNF and the microglial marker CD11b (Figure 2A), the neuronal marker NeuN (Figure 2B) or the astroglial marker GFAP (Figure 2C). Under resting conditions microglial cells expressed basal levels of BDNF and had highly ramified fine processes, but when activated by the excitotoxin, they changed to an amoeboid phagocytic-like morphology and overexpressed BDNF (Figure 2A). This can be seen in Figure 2A (Merge) as double-labelling in the lower left quadrant of the image whereas BDNF expression from other cell type(s) (presumably neurons) is apparent in the upper right quadrant of the same image. Similarly, BDNF immunoreactivity in both control and DOM-treated groups was also observed in NeuN-positive cells (Figure 2B) while only a reduced number of GFAP-positive cells expressed the neurotrophin (Figure 2C).

### DOM activates ERK1/2, PKA and CaMKII signaling pathways in hippocampal slices

**Figure 3:**
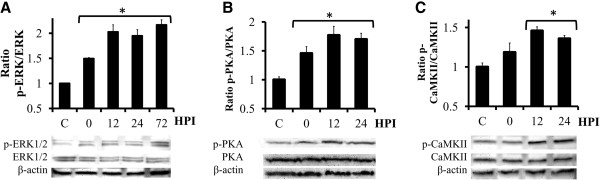
**Transient DOM exposure induces ERK, PKA and CaMKII activation in OHSC.** Cultures were treated with 2 μM DOM for 24 h (insult) and then changed to a DOM-free medium; lysates were collected at different hours post-insult (HPI) and analyzed by Western Blots for the active, phosphorylated form of ERK **(A**, p-ERK, PKA**) (B**, p-PKA**)** or CaMKII **(C**, p-CaMKII**)**. The blots were then reprobed for total ERK, PKA or CaMKII protein and finally for β-actin. The blots correspond to representative experiments and values are the means ± SEM of at least three experiments performed from different cultures (*P < 0.01 vs. C).

Next, to examine the cellular pathways activated by DOM, phosphorylation of ERK1/2, PKA and CaMKII was examined by Western blot analysis. DOM insult (2 μM, 24 h) led to an increased phosphorylation of ERK1/2 (p-ERK), with significant activation relative to baseline levels starting 0 h post-insult (HPI), reaching peak levels at 12 HPI (~2 fold) and being sustained throughout the 72 h period (Figure 3A). Phospho-PKA (p-PKA) activation was also significantly increased in OHSC following DOM insult (Figure 3B). Protein phosphorylation was significantly increased immediately following the insult (0 HPI), and reached peak expression at 12 HPI (~1.8 fold). These results indicate that both ERK and PKA reached peak activation prior to maximal increases in BDNF and TrkB receptor expression (Figure 1).

Phospho-CaMKII (p-CaMKII) levels also increased significantly over the 24 h period (Figure 3C). P-CaMKII levels were significantly increased relative to control levels with peak activation starting at 12 HPI (~1.5 fold).

### Inhibitors of MEK and PKA pathways suppress DOM-stimulated increases in BDNF expression

To examine if the ERK, the PKA or the CaMKII pathways are involved in DOM-induced BDNF overexpression in OHSC, we treated the cultures with the MAPKK (Raf)/ERK kinase (MEK) inhibitor PD98059, the PKA inhibitor H89 or the CaMKII inhibitor KN93. To confirm that CaMKII, PKA and ERK pathways are reliably inhibited by the compounds listed above at the concentration used, we measured the levels of activation of the corresponding proteins after the application of these agents. The slices were exposed to the inhibitors 1 h before DOM treatment. The results are summarized in Additional file 1. Interestingly, DOM-stimulated CaMKII activation was prevented by the MEK inhibitor PD98059 (Additional file 1). Coincubation of DOM and PD98059, but not H89 (data not shown) decreased CaMKII phosphorylation relative to that elicited by DOM. DOM-induced activation of ERK was prevented by neither KN93 nor H89 (data not shown). Hippocampal slices co-incubated with H89 or PD98059 elicited p-PKA levels that were not significantly lower than those measured by DOM alone (data not shown).

**Figure 4:**
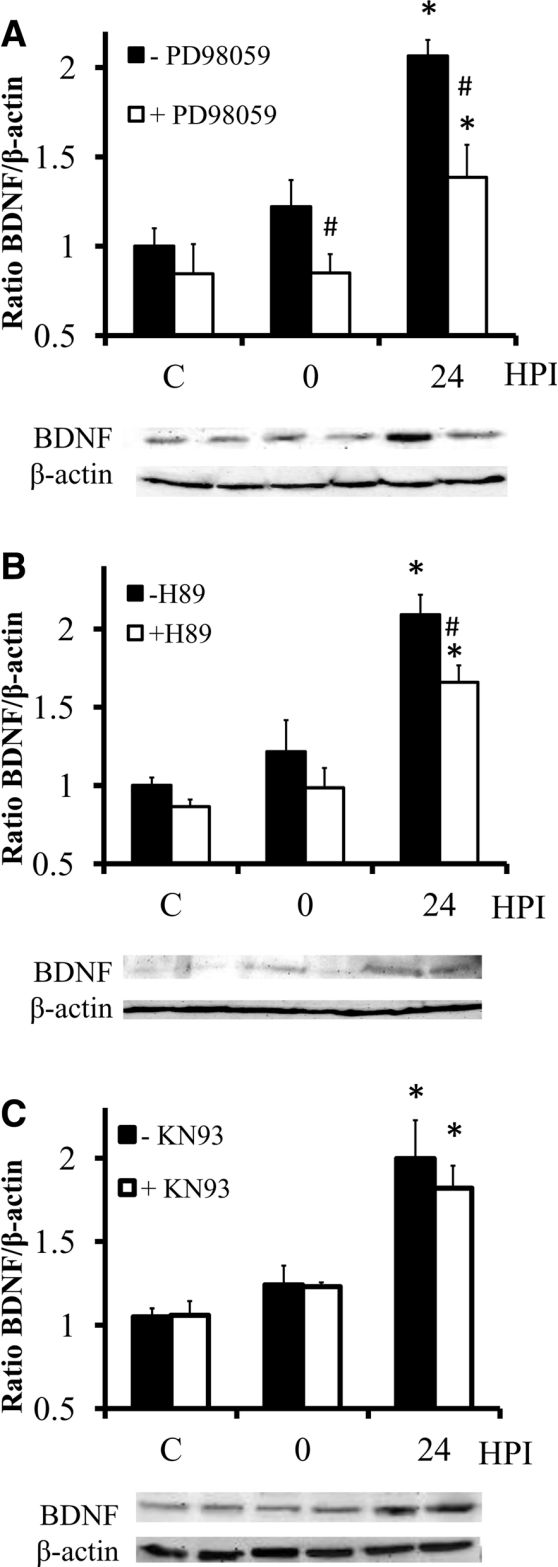
**MEK and PKA inhibition reduces the up-regulation of BDNF induced by DOM insult.** Organotypic slices were cultured in the presence of the MEK inhibitor PD98059 **(A)**, the PKA inhibitor H89 **(B)** or the CaMKII inhibitor KN93 **(C)** for 1 hour and then treated with 2 μM DOM for 24 h (insult). Lysates were collected immediately after the 24 h DOM-treatment (0 hours post-insult, HPI) or after 24 h resting in the DOM-free medium (24 HPI), analyzed by immunobloting using a BDNF antibody and the membranes were reprobed with β-actin as a loading control. The blots correspond to representative experiments and values are the means ± SEM of at least three experiments performed from different cultures (#*P* < 0.01 vs. – PD98059, H89 or KN93 within the same group; *P < 0.01 vs - PD98059, H89 or KN93 within C group).

To test whether the ERK pathway is involved in DOM-induced BDNF overexpression in OHSC the MEK inhibitor PD98059 was added to the cultured slices 1 h before DOM treatment (Figure 4A). Western blot analysis demonstrated that PD98059, when coincubated with DOM, significantly decreased DOM-stimulated upregulation of BDNF expression (~ 65% reduction). We then used a similar approach to examine the involvement of the PKA pathway on the overexpression of BDNF after DOM insult. Although not as effective as PD98059, the PKA inhibitor H89 reduced by approximately 45% the DOM-stimulated upregulation of BDNF (Figure 4B). Taken together, these results suggest that the DOM-induced rise in BDNF levels is largely both ERK and PKA-dependent. On the other hand, the CaMKII inhibitor KN93 failed to suppress or reduce the increased expression of BDNF induced by the transient injury (Figure 4C).

### DOM stimulates hippocampal CREB activation

**Figure 5:**
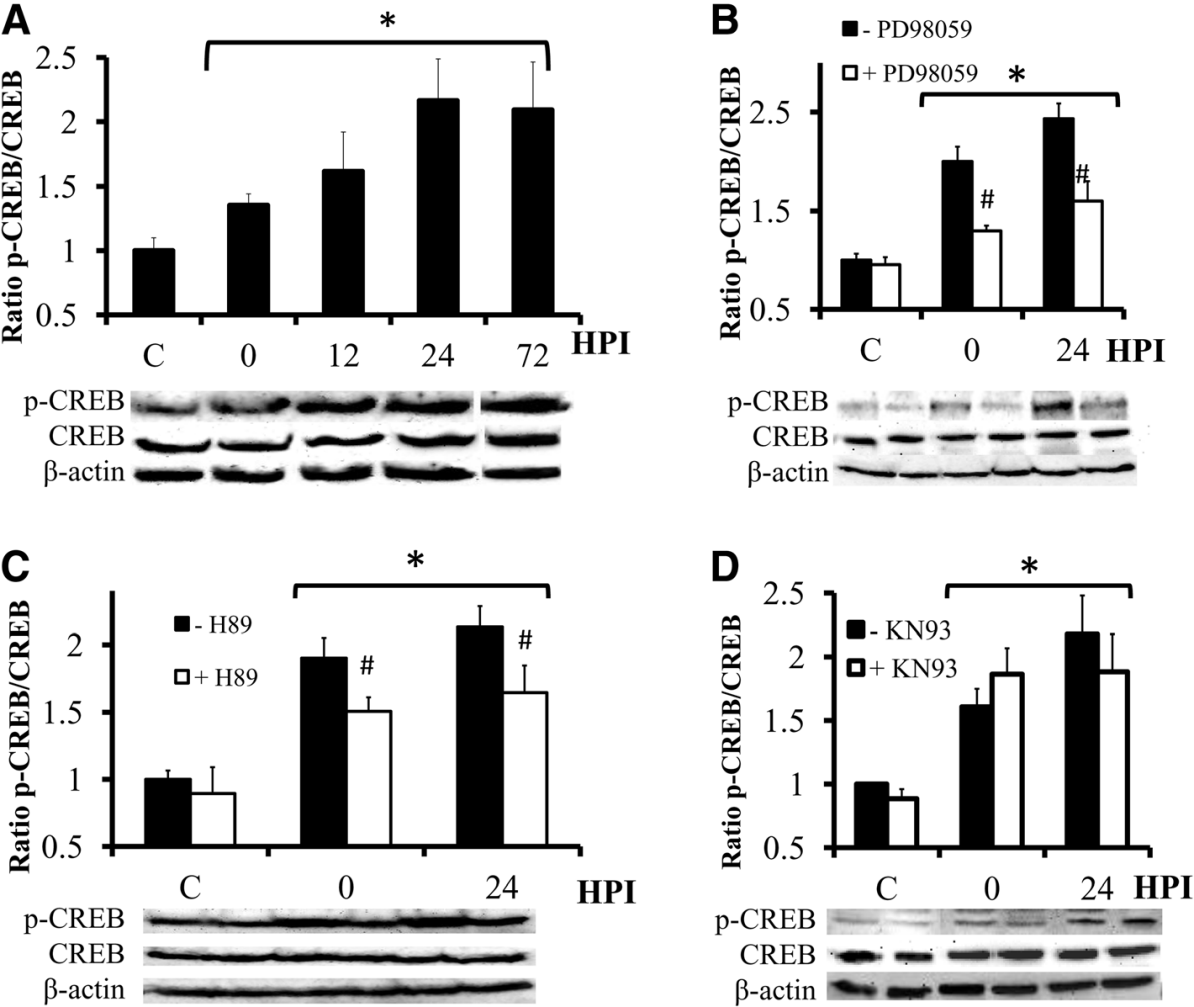
**Inhibitors of phosphorylation of the MEK and the PKA pathways reduce DOM-stimulated CREB phosphorylation. (A)** Transient DOM application (2 μM, 24 h) to hippocampal slices significantly enhanced CREB phosphorylation (*P < 0.005 vs. C). **(b**-**d)** Organotypic slices were cultured in the presence of the MEK inhibitor PD98059 **(B)**, the PKA inhibitor H89 **(C)** or the CaMKII inhibitor KN93 **(D)** for 1 h, and then treated with 2 μM DOM for 24 h (insult). Lysates were collected at the indicated hours post insult (HPI). Compared to DOM alone, the increased CREB phosphorylation was reduced when DOM was coincubated with PD98059 **(B)** or H89 **(C)**. However, KN93 did not inhibit the response to DOM (**d**). The blots correspond to representative experiments and values are the means ± SEM of at least three experiments performed from different cultures (#*P* < 0.005 vs. -PD98059, H89 or KN93 within the same group; *P < 0.01 vs - PD98059, H89 or KN93 within C group).

Both BDNF and TrkB gene expression are known to be upregulated through phosphorylation of the transcription factor CREB [35-39]. Since CREB activation has been proven to enhance hippocampal neurogenesis [40,41], as has a low concentration of DOM [7], we investigated whether phosphorylated CREB (p-CREB) was up-regulated in OHSC by DOM insult. The total amount of CREB and p-CREB in control and DOM-treated slices was determined by Western blotting (Figure 5A). Organotypic slices were exposed to 2 μM DOM and returned to DOM-free culture medium after 24 h (insult). We found that the insult increased CREB phosphorylation in a time-dependent manner. The increase was first detected immediately after termination of the DOM insult (0 hours post-insult (HPI)) and reached peak activation 24 HPI (~2 fold), remaining elevated until the end of the experiment (72 HPI).

There is ample evidence that the MAPK signaling pathway is involved in the phosphorylation of CREB to promote neuronal survival and protection [42,43]. In the current study, the MEK inhibitor PD98059 significantly decreased p-CREB levels (70% decrease) compared to the increase elicited by DOM alone (Figure 5B). The observed increase in p-CREB immunoreactivity in OHSC after DOM insult was also down-regulated when DOM was combined with the PKA inhibitor H89 (35% decrease, Figure 5C). On the other hand, when coincubated with DOM, KN93, a well-known CaMKII inhibitor, failed to block the increase in p-CREB at either time-point evaluated (Figure 5D). None of these treatments altered the protein expression of CREB.

### Neurogenesis is up-regulated via activation of both the PKA and the MEK pathway

**Figure 6:**
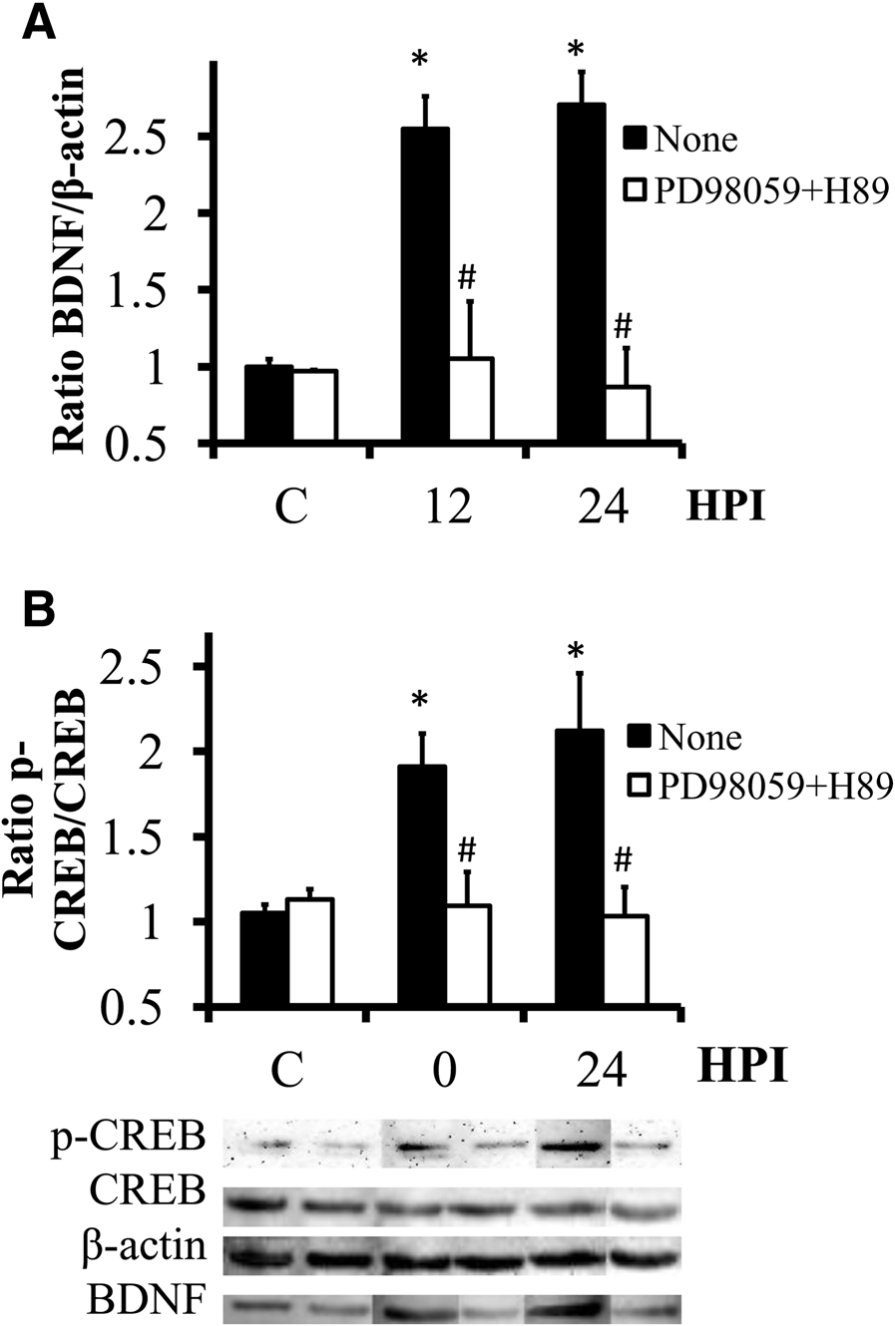
**Coexposure to MEK and PKA inhibitors suppress DOM-stimulated BDNF overexpression and CREB phosphorylation.** Organotypic slices were cultured in the presence of the MEK inhibitor PD98059 and the PKA inhibitor H89 for 1 h, and then treated with 2 μM DOM for 24 h (insult). Lysates were collected at the indicated hours post insult (HPI) and analyzed by Western blot using BDNF and β-actin antibodies **(A)** or p-CREB and total CREB antibodies **(B)**. The blots correspond to representative experiments and values are the means ± SEM of at least four experiments performed from different cultures. (#*P* < 0.001 vs. None within the same group; *P < 0.001 vs None within C group).

As described above, blocking the MEK pathway with PD98059 or the PKA pathway with H89 significantly attenuated DOM-induced overexpression of BDNF, but neither antagonist alone was able to restore immunoreactivity to control levels (Figure 4). Concurrent exposure of cultured slices to PD98059 and H89 1h before DOM treatment completely blocked the DOM-stimulated increase in BDNF expression in OHSC (Figure 6A). When PD98059 and H89 were combined with DOM, p-CREB levels were also comparable to untreated controls (Figure 6B). These data suggest that both the PKA and the ERK pathways are stimulating p-CREB phosphorylation and the subsequent production of BDNF in parallel.

**Figure 7:**
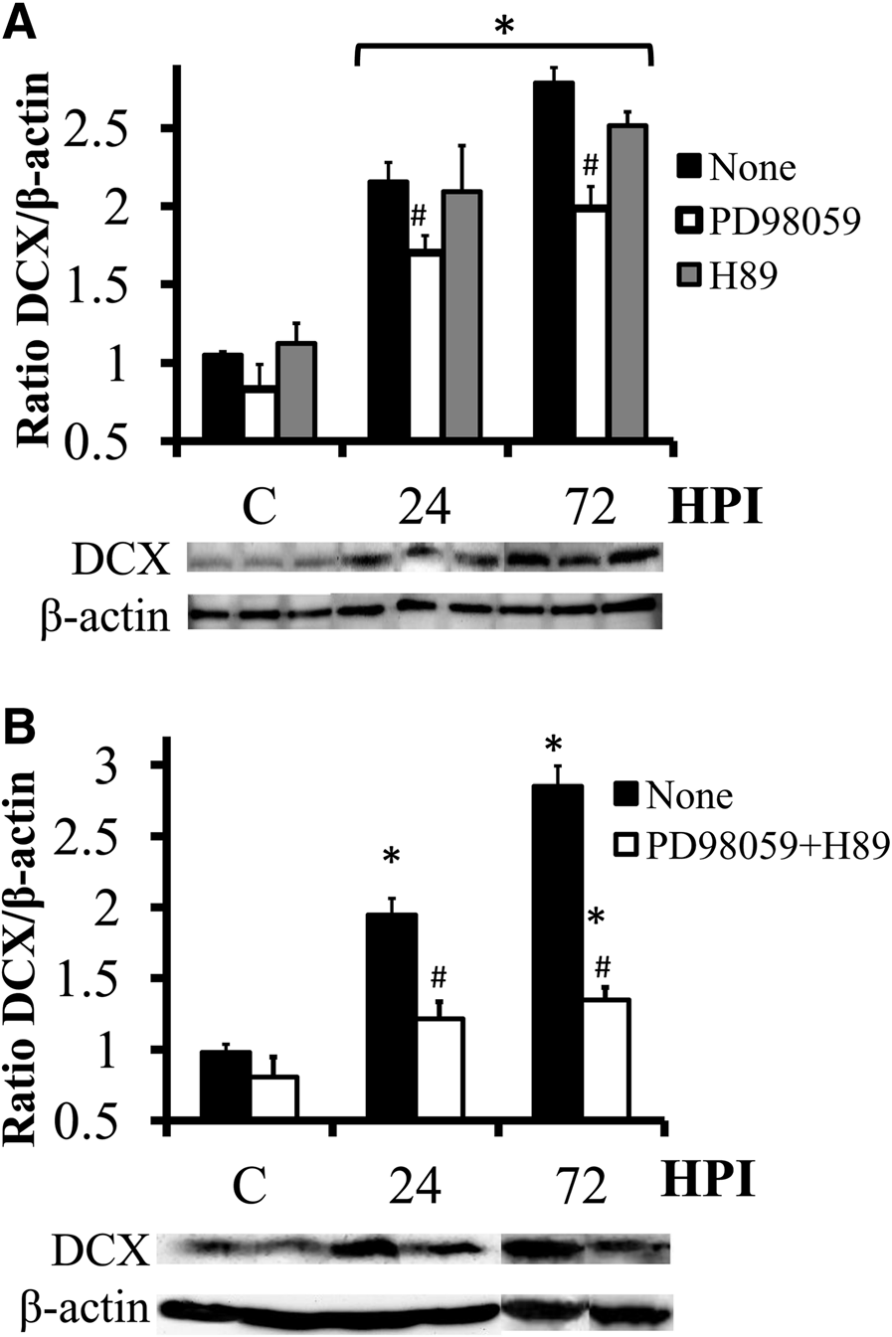
**Coexposure to the MEK and the PKA inhibitors reduces DOM-stimulated DCX upregulation. (A)** Organotypic slices were cultured in the presence of the MEK inhibitor PD98059 or the PKA inhibitor H89 for 1 h, and then treated with 2 μM DOM for 24 h (insult). Lysates were collected at the indicated hours post insult (HPI). Compared to DOM alone, the increased DCX levels were significantly reduced when DOM was co-incubated with PD98059. However, H89 failed to decrease DCX up-regulation. The blots correspond to representative experiments and values are the means ± SEM of at least three experiments performed from different cultures. **(B)** Organotypic cultures were treated with PD98059 and H89 for 1 h, and then exposed to 2 μM DOM for 24 h. Lysates were collected at the indicated HPI and analyzed by Western blot. The blots correspond to representative experiments and values are the means ± SEM of at least four experiments performed from different cultures. (#*P* < 0.001 vs. None within the same group; *P < 0.02 vs None within C group).

We have reported previously that DOM insult (24 h, 2 μM) resulted in increased neurogenesis in OHSC [7]. In order to evaluate the potential role of MEK and PKA activation pathways, OHSC were treated with PD98059 or H89 1h prior to DOM insult. We performed Western blot analysis for doublecortin (DCX), a microtubule-associated protein widely expressed exclusively in neural progenitor cells that, as we have reported previously using immunohistochemistry [7], was significant upregulated after DOM insult. Analysis of lysates revealed that DOM insult increased significantly DCX expression (Figure 7), confirming the previously published immunohistochemistry results [7]. Western blots further demonstrated that the MEK inhibitor significantly decreased the DOM-stimulated upregulation of DCX expression (~ 25% reduction). On the other hand, when coincubated with DOM, the PKA inhibitor failed to block the DCX increase (Figure 7A). Coapplication of PD98059 and H89 1 h before DOM treatment led to a greater decrease in DCX levels (Figure 7B). This additive effect suggest that PKA and ERK activate the DCX pathway independently in OHSC after DOM insult and that ERK is, to some degree, capable of compensating for the inhibition of PKA.

## Discussion

In a previous study, we demonstrated that a mild reversible injury to the hippocampal CA1 subfield induced by a low concentration of DOM increases neurogenesis in both the dentate gyrus and the CA1 subfields of OHSC [7]. Neuronal injury can lead to neural proliferation as a compensatory mechanism for cell death in the hippocampus [44,45] and growth and mitogenic factors, such as BDNF, play a prominent role in proliferation and neurogenesis after excitotoxicity [46]. In the present study, we investigated whether DOM alters BDNF expression after transient insult and explored the key intracellular signaling mechanisms by which DOM modulates neurogenesis. Our results showed that DOM insult upregulated BDNF expression by activation of both MAPK and PKA cascades and that these two pathways mediate, at least in part, the increased neural proliferation resulting after mild excitotoxicity.

Exposure to 2 μM DOM for 24 h (insult) followed by recovery induced a significant and long lasting increase in BDNF protein levels in OHSC. BDNF is a member of the neurotrophin family widely distributed in the brain with the highest levels in the hippocampus [47]. It has been previously reported that excitotoxicity and seizure activity induce an overexpression of hippocampal BDNF at both protein and mRNA levels [48-51]. BDNF signals primarily through its high-affinity receptor TrkB that promotes neurogenesis, synaptic plasticity and cell survival [52,53], and plays an important role in the development and plasticity of the brain [54]. Consistent with the observed increase in BNDF expression, DOM insult also induced TrkB upregulation. Although TrkB phosphorylation, which was not assessed in the current study, is required for receptor-mediated signaling, a number of recent papers have reported that increases in both BDNF and TrkB expression correlate with functionally-relevant downstream effects both *in vitro *[55,56] and *in* vivo [57,58]. Thus, DOM-induced changes in growth factors and/or their receptors could stimulate the increased cell birth observed after excitotoxicity.

To determine the cellular source of increased BDNF we performed double-label immunohistochemistry in the CA1 hippocampal subfield. Although the response of progenitor cells in different hippocampal regions may vary (for review see [59]) we have shown previously that the CA1 region is particularly sensitive to both excitotoxic damage by DOM and shows robust microglial activation whereas other regions (eg. SGZ) do not [7]. Our observation that BDNF is overexpressed in CA1 not only by neurons but also by microglial cells (Figure 2A) is in accordance with previous studies [13,60-62], which highlights the importance of microglial cells as a source of BDNF following injury. Examination of the image presented in Figure 2A (Merge) shows clear double-labelling of BDNF and CD11b in the lower left quadrant while cells in the upper right quadrant (presumably neurons) express only BDNF. Further, the image shows that the two cell types are in very close proximity in this region. Therefore, we suggest that under mild excitotoxic conditions both neurons and microglia will respond with an increase in the production and release of BDNF.

Clinical and basic evidence supports the idea that abnormalities in brain neuronal regeneration assisted by BDNF are associated with a wide range of disorders such as neurodegenerative diseases and psychiatric or stress-related conditions (reviewed by [63,64]). Our laboratory has reported previously that low concentrations of DOM administered *in vivo* during perinatal development cause permanent alterations in both behaviour and hippocampal structure consistent with many animal models of temporal lobe epilepsy as well as what is found in the human condition. Increased expression of both BDNF [10] and its corresponding TrkB receptor [11] were found in the hippocampus of DOM treated rats. Thus, the changes observed in OHSC in the current study are consistent with observations *in vivo*. The organotypic hippocampal slice culture system, however, provided us the means by which to evaluate the intracellular mechanism of enhanced BDNF expression initiated by transient DOM injury. Using immunobloting of specific signaling intermediates, we followed three important intracellular cascades: the MAPK, the PKA and the CaMKII pathways.

DOM insult led to increased p-ERK1/2 (p-ERK); two signaling proteins activated by the mitogen-activated protein kinase (MAPK) pathway. ERK1/2 (ERK) promote growth and modulate differentiation and survival via transcriptional regulation. ERK activation in OHSC was increased immediately following DOM exposure, reaching peak expression at 12 h post insult. DOM also caused a significant upregulation of p-PKA levels. Increases in intracellular Ca^2+^ by activation of NMDA receptors, AMPA/kainate receptors, or calcium channels increases intracellular cyclic AMP (cAMP) through activation of adenylyl cyclases that will result in the activation of PKA [65-67]. In addition to the increased p-ERK and p-PKA our results also demonstrated significant activation of CaMKII in OHSC. Other studies have reported a pivotal role for both PKA and CaMKII activation after long-lasting potentiation induced by a brief DOM treatment [68] and administration of DOM at doses that produce no major observable behavioral changes has been previously shown to increase significantly CaMKII phosphorylation [69]. Therefore, these results suggest that alterations in intracellular signaling pathways might be a protective mechanism against DOM-induced excitotoxic damage.

Ca^2+^-mediated signaling pathways tightly modulate BDNF expression mainly through the transcription factor CREB [38,70,71]. In conjunction with the observed increase in BDNF and TrkB, DOM insult was found to stimulate activation of CREB in hippocampal cultures. Several studies have proven that CREB activation requires serine-133 phosphorylation, which can be mediated by PKA, MAPK pathway or CaMKs, among others, depending on the activating signal and cell type [15-17]. In the current experiments, inhibitors of both MEK and PKA attenuated the DOM-stimulated activation of CREB as well as upregulation of BDNF. In contrast, the CaMKII inhibitor failed to prevent or significantly decrease any of the protein changes observed. These data strongly suggest that transient DOM exposure in hippocampal cultured slices upregulates CREB-dependent transcription of BDNF by activating the MAPK and PKA pathways rather than the CaMKII cascade. ERK activation has been previously associated with the transcription factor CREB in cultured hippocampal neurons and brain slices [72,73] and as MAPK signaling is required for prolonged CREB phosphorylation [74,75], it has been suggested that MAPK signalling might be highly relevant for the activation of CREB-dependent transcription. It has also been reported that PKA regulation of transcription via CREB is implicated in brain plasticity, learning and memory [76-79]. Our results showed that the DOM-induced increases in BDNF expression and CREB phosphorylation were completely blocked with concurrent exposure to PKA and MEK inhibitors. We further explored whether crosstalk between the PKA and ERK pathways might also play a role in the observed activation of CREB following DOM insult. Although evidence of coupling between these signaling pathways has been provided previously *in vivo* and *in vitro *[71,80-82] no evidence was found in OHSC after DOM insult; namely, the MEK inhibitor PD98059 failed to modulate PKA pathway activation and no significant changes were found in p-ERK levels after concurrent exposure to the PKA inhibitor H89 and DOM compared to exposure to DOM alone. Together, these pieces of evidence suggest that the PKA- and MEK-activated pathways are operating in parallel in this system and converge upon CREB, leading to BDNF overexpression. An interesting but currently unexplained finding from our experiments was that the DOM-induced increase in CaMKII was attenuated with MEK inhibition. It has been previously described that CaMKII, as an upstream kinase, interacts with Raf, modulating the activation of ERK proteins [83-85] but, to our knowledge, there is no previous evidence of ERK acting as an upstream regulator of CaMKII phosphorylation in the CNS. The observed phenomenon and its implications should be investigated further in a future study.

A major goal of this study was to elucidate the relationship between PKA/ MAPK pathways and the increased neurogenesis we reported previously in OHSC using both immunostaining and DCX positive cell counts [7]. As shown in Figure 7B, analysis by Western Blot revealed that concurrent chemical inhibition of PKA and MEK activation specifically attenuated the increase in the neuroblast cell marker DCX. In accordance with the results obtained in the present study, these kinases have been reported to mediate growth factor-induced neurogenesis and neuroprotection [18]. The extracellular signal-regulated kinase (ERK) is activated by MEK in response to growth stimuli [19] and much evidence exists that the ERK pathway plays a role in progenitor cell proliferation or differentiation in a number of model systems. For example, the ERK pathway is involved in neurogenesis, neurite outgrowth, and neuronal survival induced by either neurotrophic factors [20,21] or pharmacological agents such as valproate [22] or lithium [86] and it has been proven that ERK activation promotes hippocampal neurogenesis *in vivo *[26,27] and *in vitro*[28,29]. Similarly, PKA regulation of transcription via CREB has been associated with growth factor-dependent neurogenesis, cell survival, synaptic transmission and cognitive function in the nervous system [23-25].

Phosphorylation of CREB and overexpression of BDNF have been implicated in the regulation of the expression of many genes and cellular processes important in brain function [77] and the up-regulation of hippocampal cell proliferation [40,41]. We have previously shown that neurogenesis after DOM insult in OHSC occurred primarily during the first week of exposure in both the subgranular zone of the hippocampus and in the CA1 hippocampal subfield, with a decreasing tendency clearly observed over the next days [7]. In the current study, DOM insult induced a significant long lasting increase in BDNF protein levels in OHSC that was sustained throughout the 14 day period, although in the current study we did not determine if this effect was regionally selective. BDNF is one of the most studied extrinsic factors that not only promotes neurogenesis, but also regulates dendrite outgrowth [87-92], increases proximal dendrite growth and number in pyramidal neurons [89,91,93] and promotes synaptogenesis [94-96] and neuronal survival during development [97]. Our results suggest that BDNF in OHSC may be promoting neurogenesis as well as maturation and integration of new neurons after DOM insult, although the specific hippocampal regions at which these neurons originate, whether they in fact migrate to, or originate in, areas of transient damage, and whether they are capable of restoring normal function to the resulting circuitry needs to be determined.

## Conclusions

We have demonstrated that transient excitotoxic insult induced by DOM promotes sustained BDNF and TrkB overexpression in OHSC as well as increased hippocampal neurogenesis. Enhancement in BDNF protein levels and over-phosphorylation of its transcription factor CREB occurred via two distinct and independent signalling cascades, MEK and PKA pathways, which may be critical for hippocampal recovery after the transient DOM insult due to their role in the neurogenic process.

## Methods

### Organotypic hippocampal slice cultures (OHSC)

OHSC were prepared from 5 to 6-day-old Sprague Dawley rats (Charles River, Quebec, Canada) according to the interface method of Stoppini et al. [98]. Pups were decapitated, the brain was removed, hippocampi were dissected and transversely sliced at a thickness of 400 μm, and transferred into ice-cold dissection buffer containing 1% penincilin-streptomicin solution (Gibco, NY, USA), 25 mM HEPES (Fisher Scientific, NJ, USA) and 10 mM TRIS (Fisher Scientific) in Minimum Essential Medium (Gibco), and selected for clear hippocampal morphology (intact CA regions and dentate gyrus). The slices were transferred onto 0.4 μm porous Millicell membrane inserts (Millipore, MA, USA) and placed in individual 35 mm plates with 1 ml of serum-based medium containing 50% Minimum Essential Medium, 25% Hanks' balanced salt solution (Gibco), 12 mM HEPES, 25% heat-inactivated horse serum (Gibco) and 1% penicillin-streptomycin solution in a humidified chamber with 5% CO_2_ at 37°C. Media was changed twice a week. All animals were cared for following procedures approved in advance by the University of Prince Edward Island Animal Care Committee, and were in accordance with the Canadian Council on Animal Care guidelines. All possible efforts were made to minimize animal suffering and the number of animals used.

### DOM-induced excitotoxic injury and pharmacological treatments

At 13 days *in vitro*, damaged OHSC were excluded by propidium iodide staining (5 μg/ml for 30 min) (PI, Sigma-Aldrich, MO, USA) using a Fluoroarc exciter lamp with a Zeiss Axioplan2 microscope (Carl Zeiss Canada Ltd, ON, Canada). PI-negative slices were exposed to the indicated treatments. Cultures were exposed to DOM (2 μM, BioVectra dcl, Canada) for 24 h and then transferred to a DOM-free medium. The MEK inhibitor PD98059 (20 μM, Cell Signaling, MA, USA), the PKA inhibitor H89 (10 μM, Calbiochem, MA, USA) as well as the CaMKII inhibitor KN93 (10 μM, Sigma) were added to the culture medium 1 h before DOM and maintained throughout the experimental period.

### Immunohistochemistry

Cultures were washed in 0.1 M phosphate-buffered saline (PBS), fixed in formalin for 18 h and cryoprotected in 30% sucrose/PBS for an additional 18 h. OHSC were then further sliced into 15 μm sections on a cryostat, mounted on glass slides and stored at − 20°C. After culturing for up to 4 weeks OHSC thin down from the original 400 μm to about 180 μm. For cryosectioning the first two sections of 15 μm were discarded since this part contains the glial scar [99]. For immunohistochemistry the next 4–5 15 μm cryosections were saved which resulted in collection of the middle part of each hippocampal slice culture. The following primary antibodies were used: mouse anti-NeuN (1:250, Millipore #MAB377), mouse anti-GFAP (1:400, Sigma #G9269), mouse anti-CD11b (1:100 AbD serotec, NC, USA #MCA275R) and rabbit anti-BDNF (1:100, Millipore #AB1779). The following secondary antibodies were used: Alexa Fluor 488 goat anti-rabbit IgG (1:200, Invitrogen, OR, USA #11034) and Alexa Fluor 594 goat anti-mouse IgG (1:200, Invitrogen #11032). Negative controls (i.e. buffer substitution) for all primary and secondary antibodies were included in every run and displayed no specific staining at any time. For double immunostaining, cryosections were washed in PBS, blocked with 3% normal goat serum (Vector Laboratories Inc. CA, USA) and 0.5% bovine serum albumin (BSA) in PBS mixed with 0.1% Triton for 1 h at room temperature and incubated with the indicated primary antibodies in 2% goat serum /PBS + 0.1% Triton overnight. After rinsing in PBS, sections were incubated with the corresponding secondary antibodies for 1 h and washed 4 times. Slices were incubated with 300 nM DAPI in PBS for 2 min, washed and mounted with Citifluor (Canemco-Marivac, P.Q, Canada). Images from 12 to 15 cryoslices from 3 different preparations were acquired using a Zeiss Axioplan 2 microscope and digital Axiocam camera. AxioVision software was used to standardize the images by setting all the parameters to a constant value.

### Western blotting

Slice cultures were collected and homogenized on ice in a lysis buffer (63 mM Tris HCl, 10% SDS, 2 mM EDTA) mixed with phosphatase inhibitor cocktail tablets (Roche Diagnostics, IN, USA) and complete protease inhibitor mix (Roche Diagnostics). Protein concentration was determined using the BCA protein assay kit (Thermo Scientific, IL, USA). Samples were heated to 95°C for 5 min, and equal quantities of protein extract (20 μg) were separated on 12% SDS gels. Proteins were transferred to polyvinylidene difluoride membranes (Bio-Rad Laboratories Inc., CA, USA #1620177) and incubated with specific antibodies raised against BDNF (Millipore #AB1779), CREB (Cell Signaling #9197), phosphorylated CREB (Cell Signaling #9198S), CaMKII (Santa Cruz Biotechnology, CA, USA #sc5306), phosphorylated CaMKII (Santa Cruz Biotechnology #sc32289), ERK (Cell Signaling #9102), phosphorylated ERK (Cell Signaling #9106), PKA (Santa Cruz #sc365615), phosphorylated PKA (Santa Cruz #sc32968), DCX (Santa Cruz Biotechnology #sc32968) and TrkB (Santa Cruz Biotechnology #sc12). The BDNF antibody reacts against mature BDNF (14 KDa) as well as pro-BDNF (28 KDa). Results shown in this study correspond to the 14 KDa band. A control for protein loading was performed by reprobing membranes with an antibody against β-actin (Sigma-Aldrich). No significant changes during the 2 weeks culture period without drug treatment were observed for any of the measured proteins. Membranes were incubated with secondary anti-mouse or anti-rabbit IgG Peroxidase (Sigma-Aldrich). Immunopositive bands were visualized using the Enhanced Chemiluminescence Plus western blotting system from Amersham (UK). Pictures of the bands were taken and a subsequent analysis was performed on a Biospectrum AC Imaging System (UVP, Upland, CA, USA) using VisionworksLS software (v 6.7.4, UVP). Values obtained were normalized and expressed as the ratio obtained from cultures under control conditions.

### Statistical analysis

All data are given as mean ± SEM and statistical significance was evaluated by One-Way ANOVA followed by Tukey's post hoc test using GraphPad Prism 5.0. *P* < 0.05 was used as a limit for statistical significance.

## Abbreviations

AMPA: α-amino-3-hydroxy-5-methylisoxazole-4-proponic acid; BDNF: Brain derived neurotrophic factor; CA: Cornu ammonis; CaMKs: Ca^2+^/calmodulin-dependent protein kinases; CD11b: Cluster of differentiation molecule 11b; CNS: Central nervous system; CREB: Cyclic AMP responsive element binding protein; DAPI: 4',6-diamidino-2-phenylindole; DCX: Doublecortin; DI: Day of insult; DIV: Days in vitro; DG: Dentate gyrus; DOM: Domoic acid; DPI: Days post-insult; GFAP: Glial fibrillary acidic protein; HPI: Hours post-insult; MEK: Mitogen-activated protein kinase kinase; NeuN: Neuron specific nuclear protein; NMDA: N-methyl-D-aspartate; OHSC: Organotypic hippocampal slice cultures; PBS: Phosphate-buffered saline; PI: Propidium iodide; PKA: cAMP-dependent protein kinase; PND: Postnatal day; SGZ: Subgranular zone; TrkB: Tropomyosin-related kinase B.

## Competing interests

The authors have no competing interests to declare in relation to this manuscript.

## Authors’ contributions

Both authors were involved with the design of the research. APG performed research and analysed data; APG and RAT prepared the manuscript. Both authors read and approved the final manuscript.

## Supplementary Material

Additional file 1 **Specific inhibitors block the activation of their respective intermediates.** (A) PD98059 was applied to the slices 1 h prior DOM and p-ERK, ERK and β-actin levels were determined by Western Blot 24 hours post insult (HPI). (B) H89 was applied to the slices 1 h prior DOM and p-PKA, PKA and β-actin levels were determined by Western Blot 24 HPI. (C) KN93 was applied to the slices 1 h prior DOM and p-CaMKII, CaMKII and β-actin levels were determined by Western Blot 24 HPI. Blots correspond to representative experiments and values are the means ± SEM of at least three experiments performed from different cultures (#*P* < 0.001 vs. -PD98059, H89 or KN93 within the same group; *P < 0.001 vs - PD98059, H89 or KN93 within C group). (D) MEK inhibitor reduces de up-regulation in CaMKII activation. PD98059 was applied to the slices 1 h prior DOM and p-CaMKII, CaMKII and β-actin levels were determined by Western blot right after DOM exposure or 24 HPI. Blots correspond to representative experiments and values are the means ± SEM of four experiments performed from different cultures (#*P* < 0.001 vs. -PD98059 within the same group; *P < 0.005 vs - PD98059 within C group). Click here for file
