# Supplementary figures and images for: Transient domoic acid excitotoxicity increases BDNF expression and activates both MEK- and PKA-dependent neurogenesis in organotypic hippocampal slices
Source: BMC Neurosci. 2013 Jul 17;14:72. doi: 10.1186/1471-2202-14-72 (PMC3722092; doi:10.1186/1471-2202-14-72)

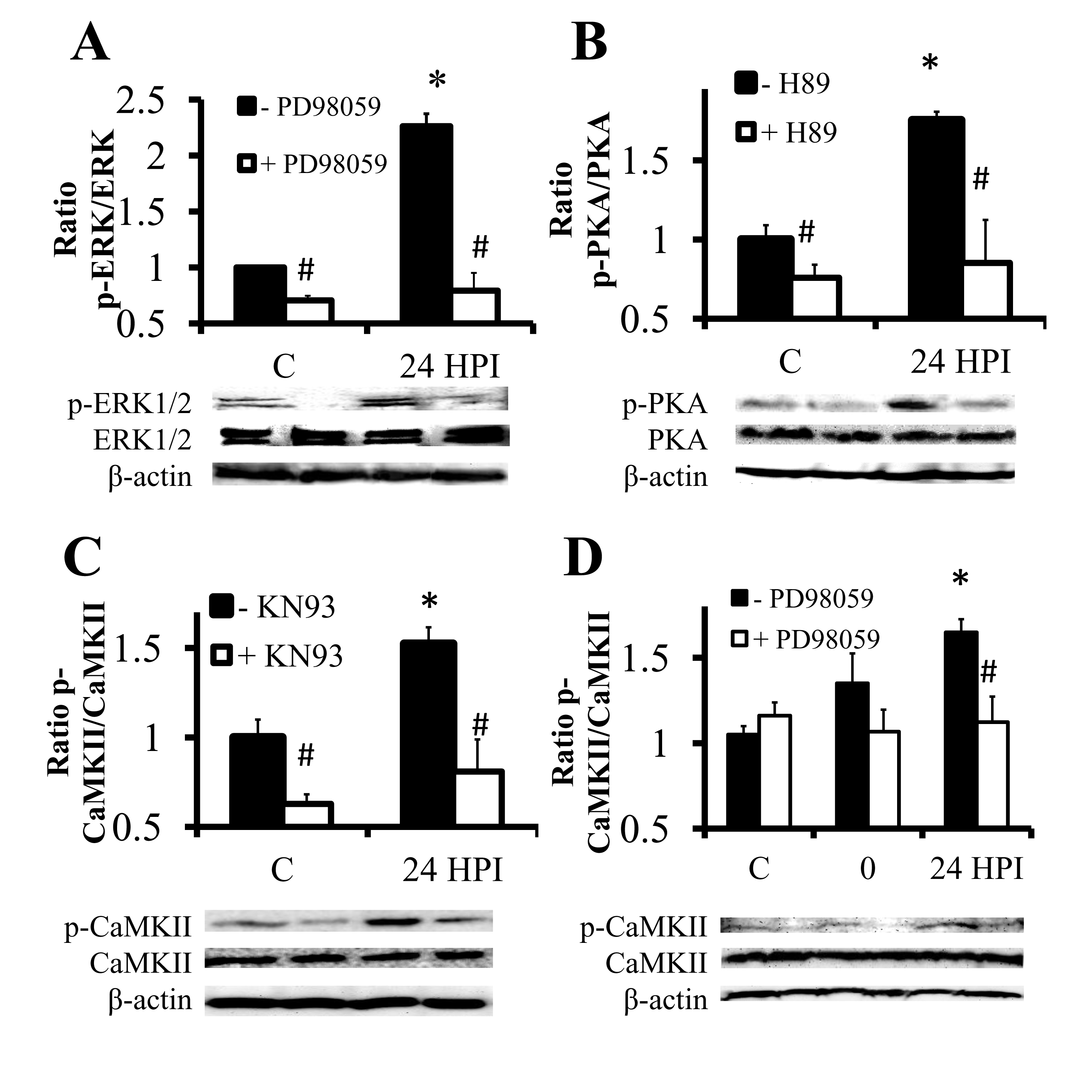

Supplement: Additional file 1 — Specific inhibitors block the activation of their respective intermediates. (A) PD98059 was applied to the slices 1 h prior DOM and p-ERK, ERK and β-actin levels were determined by Western Blot 24 hours post insult (HPI). (B) H89 was applied to the slices 1 h prior DOM and p-PKA, PKA and β-actin levels were determined by Western Blot 24 HPI. (C) KN93 was applied to the slices 1 h prior DOM and p-CaMKII, CaMKII and β-actin levels were determined by Western Blot 24 HPI. Blots correspond to representative experiments and values are the means ± SEM of at least three experiments performed from different cultures (#P < 0.001 vs. -PD98059, H89 or KN93 within the same group; *P < 0.001 vs - PD98059, H89 or KN93 within C group). (D) MEK inhibitor reduces de up-regulation in CaMKII activation. PD98059 was applied to the slices 1 h prior DOM and p-CaMKII, CaMKII and β-actin levels were determined by Western blot right after DOM exposure or 24 HPI. Blots correspond to representative experiments and values are the means ± SEM of four experiments performed from different cultures (#P < 0.001 vs. -PD98059 within the same group; *P < 0.005 vs - PD98059 within C group). [file 1471-2202-14-72-S1.tiff]
